# Bilateral Adductor Nerve Palsy Following Total Thyroidectomy: A Case Report

**DOI:** 10.31729/jnma.4253

**Published:** 2019-04-30

**Authors:** Prashant Bhatt, Apar Pokharel

**Affiliations:** 1 Department of Otorhinolaryngology and Head and Neck Surgery, College of Medical Sciences, Bharatpur, Chitwan, Nepal

**Keywords:** *palsy*, *recurrent laryngeal nerve*, *total thyroidectomy*

## Abstract

Recurrent Laryngeal Nerve palsy following thyroidectomy is usually attributed to the surgery whereas sometimes the cause can be non-surgical and can result in adductor palsy. Bilateral Recurrent Laryngeal Nerve paralysis is rare complication of thyroidectomy. We present a case of a 35 year old female who developed dysphonia following thyroidectomy. The clinical findings and recovery were suggestive of a non-surgical cause for the palsy. The management of these patients differs and the knowledge in this regard is very important for the surgeons. The non-surgical and surgical cause of adductor palsy differs in presentation and management. Tracheostomy is not required and recovery of the nerve occurs in most cases.

## INTRODUCTION

Bilateral Recurrent Laryngeal Nerve (RLN) paralysis is an uncommon complication of thyroidectomy (seen in 0.4%), the commonest surgery that puts normally functioning laryngeal nerves at risk of injury.^1,2^ Injury to the RLN could be temporary or permanent, unilateral or bilateral. Recent study showed that the likelihood of temporary RLN paralysis is higher in bilateral near total thyroidectomy compared to bilateral total thyroidectomy.^3^ Bilateral RLN paralysis is a major risk factor for dysphonia, airway obstruction and is significantly associated with post- thyroidectomy vocal cord paralysis and long term risks of hospital readmission, dysphagia, hospitalization for lower respiratory tract infection and tracheostomy/gastrostomy.

## CASE REPORT

We present a case of 35-year-old female who came to the out-patient department with a complaint of swelling in the anterior aspect of neck for 6 months. A thorough workup was done.

USG revealed a swelling of 15x5x3 mm in the right lobe of thyroid and a swelling of 1.5x1.5x1 mm in the left lobe of thyroid. FNAC revealed it to be papillary carcinoma thyroid. Thyroid function test and serum calcium levels were within normal range. Intraoperative course was uneventful.

**Figure 1.**
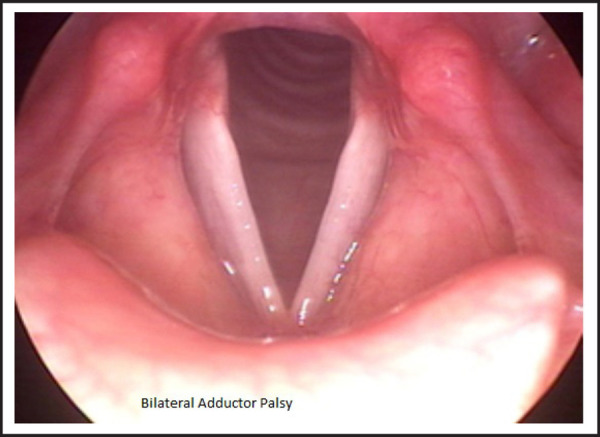
NPL showing bilateral adductor vocal cord palsy.

On first post-operative day the patient developed aphonia and on second post-operative day she developed an attack of tetany. Hypocalcemia was managed aggressively with calcium gluconate, oral calcium and vitamin D_3_. Serum calcium and magnesium levels were monitored and supplemented accordingly. Nasopharyngolaryngoscopy (NPL) was done which showed that the patient had developed a bilateral adductor palsy of the vocal cords (Figure 1).

Patient was discharged after the calcium level was normal and there were no features of hypocalcemia. NPL was done at discharge for aphonia again and same results as before were obtained. Patient was called for a follow up after speech therapy. Patient also received radioiodine therapy.

Some improvement in voice was noticed and NPL revealed mild flickering movement of the vocal cords at follow up in 4 weeks. There were no features of hypocalcemia at this time.

**Figure 2.**
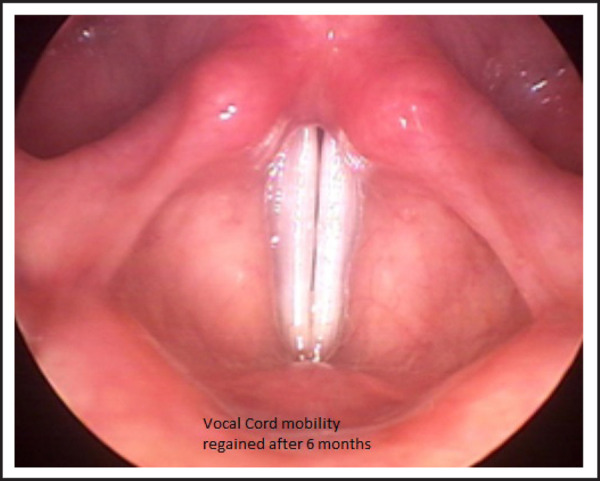
NPL showing spontaneous adduction of both vocal cords 3 months after surgery.

There was a significant improvement in voice and NPL revealed vocal cords to be mobile at 12 weeks time (Figure 2).

## DISCUSSION

The anterior branch of the RLN supplies the adductor muscles of the vocal cord, injury of which leads to the abduction of the vocal cords which leads to hoarseness of voice and aspiration but shouldn't compromise the airway. As the airway is patent tracheostomy is not required in these patients. Knowledge in this regard decreases morbidity associated. Recovery of the function occurs usually, provided there is no permanent damage due to ischemia.

Vocal cord dysfunction due to injury of the laryngeal nerve is an important complication to have knowledge about in case of thyroid surgery, the consequences can be extremely dangerous. Efforts should be taken by surgeons to limit such occurrences through a thorough understanding of the surgical anatomy, the use of loupes magnification and intra-operative nerve monitoring. The reported incidence of bilateral vocal cord palsy remains very low, ranging from 0.58 to 0.63%.^4,5^ The British Association of Endocrine and Thyroid Surgeons (BAETS) recommend that surgeons should be performing a minimum of 20 thyroidectomies per year to be considered safe^6^ and Adam et al. (2017) concluded that surgeons performing more than 25 per year had better patient outcomes.^7^ In the literature to date, there have been no reports of bilateral vocal cord palsy following hemithyroidectomy.

Non-surgical causes of vocal cord dysfunction are well known and include hypocalcaemia, low vitamin D levels^8^ and as a side effect of Cisplatin and vinca alkaloid based chemotherapy agents.^9^ Hypocalcaemia which is seen more frequently following total thyroidectomy is a well-known complication.^10^ In the present case study, the post-operative blood work up including corrected calcium, vitamin D and magnesium were with in normal range.

Numerous case reports in the literature show RLN dysfunction following endotracheal intubation due to compression.^11,12^ Cavo (1985) found 6 cases of postoperative bilateral vocal cord palsy in the literature (prior to 1985), none of which were following thyroid surgery.^13^ Using cadaveric models to investigate the mechanism of injury, he concluded that compression of the anterior branch of the nerve occurred against the rigid thyroid lamina, 6–10 mm below the posterior end of the free edge of the vocal cord. He also investigated the fluctuation in cuff pressure depending on the gas used to inflate the cuff, and found that pressure in cuffs inflated with air as in our routine practice increased by 8–10 mmHg due to diffusion of nitrous oxide across the cuff membrane. Although not routine practice, if inflated with anaesthetic gases, he found little fluctuation in cuff pressure throughout the procedure. He also suggested various measures to prevent problems associated with the increased endotracheal cuff pressure. Nuutinen et al. (1981) and Vyshnavi et al. (2013) concluded that overextension of the neck during intubation resulted in stretching of the vagus nerves. In our case, the patient was positioned with her neck extended, with a shoulder roller and head ring in position.^13,14^

## Consent

**JNMA Case Report Consent Form** was signed by the patient and the original is attached with the patient's chart.

## Conflict of Interest

**None.**
